# Geoid Undulation Model as Vertical Reference in Indonesia

**DOI:** 10.1038/s41597-024-03646-w

**Published:** 2024-07-26

**Authors:** Arisauna Pahlevi, Agustina Syafarianty, Susilo Susilo, Yustisi Lumban-Gaol, Widy Putra, Bagas Triarahmadhana, Brian Bramanto, Raa Muntaha, King El Fadhila, Febriananda Ladivanov, Harka Amrossalma, Lukman Islam, Dwi Novianto, Safirotul Huda, Tunjung Wismadi, Joni Efendi, Alkindi Ramadhan, Dudy Wijaya, Kosasih Prijatna, Gatot Pramono

**Affiliations:** 1 Directorate for Geospatial Reference System, Geospatial Information Agency, Bogor, Indonesia; 2 National Research and Innovation Agency, Bogor, Indonesia; 3 Geodetic Science, Engineering, and Innovation Research Group, Faculty of Earth Sciences and Technology, Institut Teknologi Bandung, Bandung, Indonesia

**Keywords:** Solid Earth sciences, Natural hazards

## Abstract

Indonesia released a new regional geoid model in 2020—the Indonesian Geoid 2020 (INAGEOID2020). It covers the Indonesian region with a spatial resolution of 0.01 × 0.01 degree with the unit in meters. The model was generated through a series of data and computations. Three components of gravity data, i.e., the observed free-air anomaly, the long-wave from the global geoid model, and the short-wave from the terrain model, were employed. The computation was performed using the Remove-Compute-Restore technique with the Fast Fourier Transformation approach. The output was then fitted to the geoid at tide stations by adding a fitting plane to the geoid model. The fitting plane was constructed based on the difference between the geoid model and each tide gauge benchmark. The final geoid model was evaluated by comparing the model with the reference data. Based on quality metrics, the accuracy of INAGEOID2020 varied between 6 cm to 29 cm. Any interested user can use this gridded geoid model to convert geodetic to orthometric heights and vice versa.

## Background & Summary

A datum is a realisation of a coordinate system to define a coordinate surface; a vertical datum is a referenced coordinate surface for vertical positions, such as heights or elevations^1^. Common types of vertical datums include geoid and reference ellipsoid. The geoid is one of the fundamental concepts. Among many definitions of the geoid^2–9^, it can be understood as the equipotential surface of the Earth’s gravity field, which depends upon the irregular distribution of masses of the Earth, where the surface closely coincides with the undisturbed mean sea level. It is used as a reference for orthometric heights. However, the irregular shape of the geoid makes it mathematically complex to model^9,10^. Therefore, a reference ellipsoid is commonly used instead of the geoid to represent the Earth in a mathematical model^9,11^. The reference ellipsoid is used to determine geodetic heights, which are heights provided by the Global Navigation Satellite System (GNSS). However, geodetic heights do not correctly represent the actual condition and hence cannot be used in practice, e.g., for engineering purposes^1^. Thus, practical matters on the surface require a proper geoid model to provide accurate orthometric heights.

Since both references do not coincide, the orthometric and geodetic heights differ inconsistently. These differences are called the geoid undulation. The orthometric height (*H*), geodetic height (*h*), and geoid undulation (*N*) are related to each other and expressed as

1 $$H\approx h-N\mathrm{.}$$

The equation demonstrates that a geoid undulation model can convert geodetic to orthometric heights. It will benefit height measurement using GNSS or other instruments that use reference ellipsoids to measure the height. Although global geoid models are available, they are still insufficient due to their accuracy^12^. Accordingly, many countries have developed their national geoid model to overcome such issues^13–26^. The geoid information is essential in national development planning and disaster mitigation, e.g., flood modelling and prediction^27,28^.

Indonesia is an archipelago country consisting of five big islands with thousands of small islands surrounded by the Indian Ocean, the South China Sea, the Philippines Sea, and the Pacific Ocean. The first regional geoid model in Indonesia was released in 1996, namely the Indonesian Geoid Model 1996 (INDGED96). It was obtained by combining gravity data collected through the Indonesian Gravity Database 1994 (Basisdata Gayaberat Nasional—BGN) and the Ohio State 1991 Geopotential and Sea Surface Topography Harmonic Coefficient Models (OSU91A)^29^. Compared to the preliminary model developed in 1981 with a considerably low accuracy of approximately 4 m^30^, the accuracy of the INDGED96 model improved to 1.5 m in Sumatra and 1 m in Java based on Global Positioning System (GPS) levelling validation. Despite the achievement in producing the national geoid data, INDGE96 has several limitations due to the lack of BGN data distribution in Kalimantan, Sulawesi, and Papua. In addition, the unidentified vertical datum of position data and the unreliability of the accuracy of gravity at sea derived from interpolated altimetry data urge the model to be improved to obtain a more accurate geoid model^31^. Consequently, efforts to improve the model continue by acquiring gravity data with equitable distribution and acceptable quality.

In 2020, Indonesia released a new geoid model, the Indonesian Geoid 2020 (INAGEOID2020), and continuously updated its quality through data acquisition. It differs from the previous model since it was determined using more data measurements, including airborne gravity data. INAGEOID2020 covers the Indonesian region with a spatial resolution of 0.01 × 0.01 degree (roughly 1.11 km) with the unit in metres. It complements the national datum, the Indonesian Geospatial Reference System 2013 (Sistem Referensi Geospasial Indonesia 2013—SRGI2013)^32^ by providing the reference geoid undulation. The gravity reference frame of INAGEOID2020 refers to the Geodetic Control Network (Jaring Kontrol Geodesi—JKG) pillars constrained to the International Gravity Standardization Net 1971 (IGSN71)^33^ or its upgrade version, including the International Gravity Reference System (IGRS)^34^. The model was generated through a series of computations. This paper describes how the Indonesian geoid undulation model was computed. It can be used to convert heights between the above-mentioned vertical datums.

### Observations specifications

As one of the data sources for generating the gridded geoid model, the gravity field measurements were acquired through multiple platforms, including terrestrial and airborne. Data acquisition was conducted by the Geospatial Information Agency of Indonesia (BIG) from 2008 to 2021. The terrestrial gravity was measured on the main pillars of gravity (GBU) and the JKG pillars using the Micro-G A10, Scintrex CG-6, and Scintrex CG-5. Those benchmarks are distributed over big cities with a grid distance of ~5 km (see Figure S1-S8).

Meanwhile, airborne gravity surveys were performed on five main islands, i.e., Sumatra, Java, Kalimantan, Sulawesi, Maluku, and part of Nusa Tenggara Barat, with an average flight strip distance of ~15 km (see Figure S9). The surveys were carried out using three different gravimeters, i.e., La Coste & Romberg Air-Sea Gravity S-99, owned by Danish Technical University (DTU) Denmark; La Coste & Romberg Air-Sea Gravity S-130, owned by Taiwan; and La Coste & Romberg Air-Sea Gravity GT-2A, owned by BIG.

Each measured gravity was processed using software corresponding to the instrument to retrieve free-air anomaly (FAA) as the observed gravity component. The Geodetic Gravity Field Modelling Programs (Gravsoft)^35^ computed FAA gravity from terrestrial surveys. The Anomaly Gravity Reduction (AGR) software developed by the National Chiao Tung University (NCTU)^18^ accommodated gravity data acquired by La Coste & Romberg Air-Sea Gravity S-99 and S-130. Meanwhile, Geosoft software processed data obtained by La Coste & Romberg Air-Sea Gravity GT-2A.

Following the processing analysis, the root mean square error (RMSE) for terrestrial FAA varies between 0.01 mGal and 0.895 mGal. For airborne FAA, the RMSE falls within the range of 1.5 mGal to 4.6 mGal. Additionally, a downward continuation (DWC) procedure was applied to the airborne data to derive FAA values at ground level.

## Methods

**Fig. 1:**
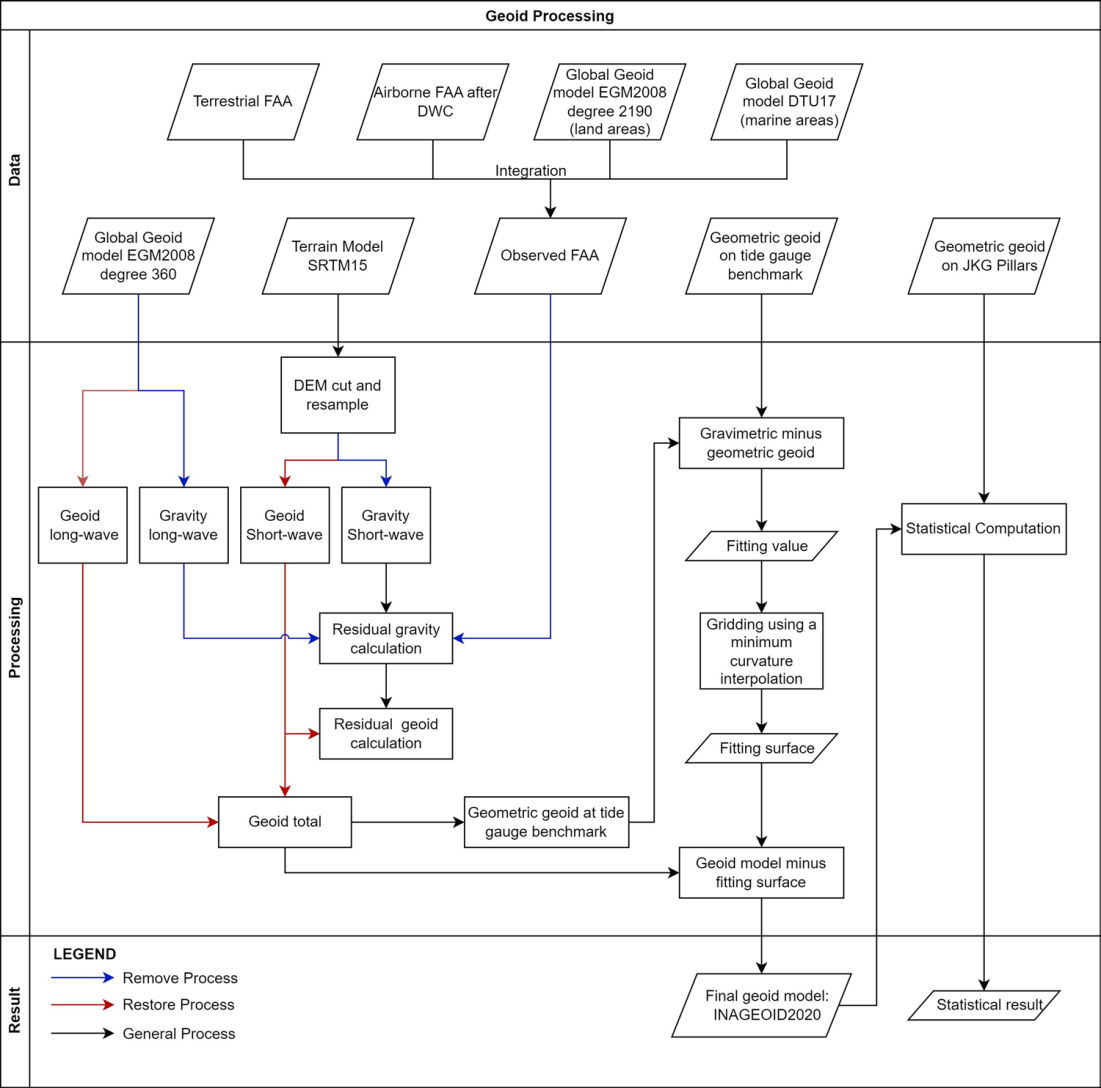
The computation procedure to generate INAGEOID2020.

INAGEOID2020 was computed using the Remove-Compute-Restore technique^36–38^ with the Fast Fourier Transformation (FFT) approach^39^. Figure 1 describes the overall process of building the model, including the data used, the processing steps, the result, and validation. This work used five datasets to generate a seamless national geoid model and to evaluate its quality, including observed FAA, long-wave component, short-wave component, geometric geoid value at each tide gauge benchmark, and geometric geoid value at JKG pillars.

**Fig. 2:**
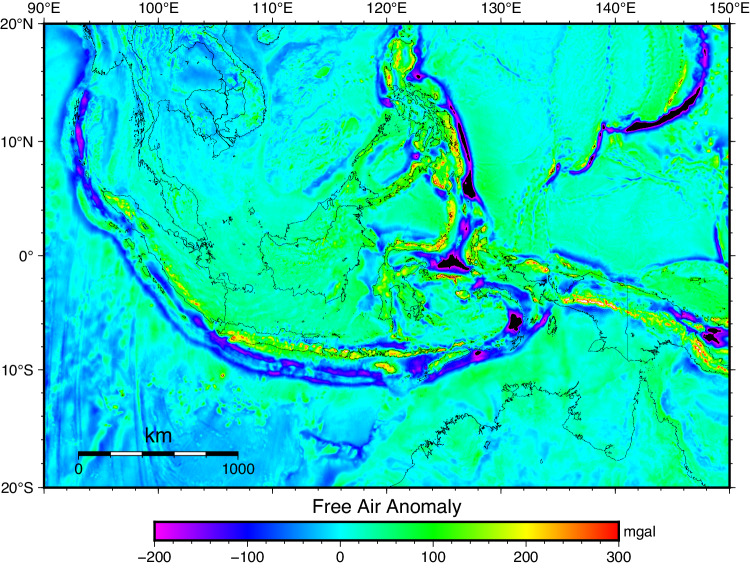
The FAA gravity of Indonesia ∆*g*_*FA*_ in mGal.

First, the observed FAA gravity (∆*g*_*FA*_) shown in Fig. 2 served as the primary data. It was produced by integrating the terrestrial, airborne, Earth Gravitational Model (EGM2008) degree 2190^40^, and DTU17 global marine gravity data^41^ (see Figure S1-S11 for all data distribution). The DTU17 data was used to fill water areas, while the EGM2008 degree 2190 aims to fill data gaps in land areas^42^. The hierarchy of those data was terrestrial, airborne, and global models. So, when DTU17 overlapped with the airborne, the FAA airborne was chosen. Similarly, the FAA terrestrial was selected when airborne data overlaid with terrestrial data. The integration process was carried out using QGIS^43^ software.

The second and third data were the EGM2008 degree 360^44^ for long-wave components and the global terrain model Shuttle Radar Topography Mission 15 m (SRTM15)^45^ for short-wave components. The EGM2008 degree 360 provides long wave gravity values in the spherical harmonic format. This input was converted into the long-wave gravity anomaly and the long-wave component geoid model. The long-wave gravity anomaly ranges from −250 to 250 mGal, while the long-wave geoid model ranges from −80 to 100 m. On the other hand, the SRTM15 data, which represents the terrain’s surface of an area with tight interval values, was converted into short-wave gravity anomaly and the short-wave geoid model using TCFOUR on Gravsoft software, which employed FFT convolutions in planar approximation^35^.

The INAGEOID2020 modelling process was accomplished using Gravsoft software, which has been modified to comply with archipelago countries like Indonesia. The process generally started by obtaining all gravity anomaly components and geoid undulation needed to produce the final geoid model. The gravity anomaly components include the observed (∆*g*_*FA*_), the long-wave (∆*g*_*GM*_), and the short-wave (∆*g*_*h*_). Meanwhile, the geoid components include the residual geoid (*N*_*res*_), the long-wave (*N*_*GM*_), and the short-wave (*N*_*h*_). The geoid was determined by applying the Remove-Compute-Restore technique, which integrated the Stokes formula through mass topography regulation^36^.

This technique removes topographic effects on the long-wave and short-wave surfaces from the observed gravity anomaly (Eq. 2) and restores those components when computing the final geoid (Eq. 3).

2 $${\triangle g}_{{res}}={\triangle g}_{{FA}}-{\triangle g}_{{GM}}-{\triangle g}_{h}$$

3 $$N={N}_{{res}}+{N}_{{GM}}+{N}_{h}$$

The observed gravity anomaly (∆*g*_*FA*_), which contains all components of gravity anomaly, is subtracted by long-wave (∆*g*_*GM*_) and short-wave (∆*g*_*h*_) components of gravity anomaly to produce the residual gravity anomaly (∆*g*_*res*_), where the result varies between −50 and 40 mGal. Using the Stokes formula^46^, the residual gravity anomaly was processed to become the residual geoid (*N*_*res*_), which ranges from −10 to 10 m. Finally, the total gravimetric geoid (*N*) was produced by enumerating all geoid components: the residual (*N*_*res*_), the long-wave (*N*_*GM*_) and the short-wave (*N*_*h*_).

The total gravimetric geoid and the geometric geoid at tide gauge benchmarks do not perfectly coincide due to sea level topography^47^ and errors in the global geoid model^48^. Therefore, the total gravimetric geoid was fitted to the geometric geoid at tide gauge benchmarks to reduce the constant offset, represented by the zero-degree term, that aligns the adopted sea level or equipotential surface to the reference ellipsoid. To fit the geoid, the differences between gravimetric and geometric geoids at selected tide gauge stations were used to construct the fitting surface. We selected tide gauge benchmarks that had a minimum of four years’ worth of ocean tide measurements and where the difference in geoid height between the tide gauge benchmark data and the corresponding geoid height from the EGM2008 geoid model was within the threshold of ±1 meter. There were 94 selected stations (see Figure S12) used to construct the fitting surface. The difference between the gravimetric and geometric geoid in each tide gauge benchmark was gridded using a surface with a minimum curvature interpolation approach, thereby forming the fitting surface.

**Fig. 3:**
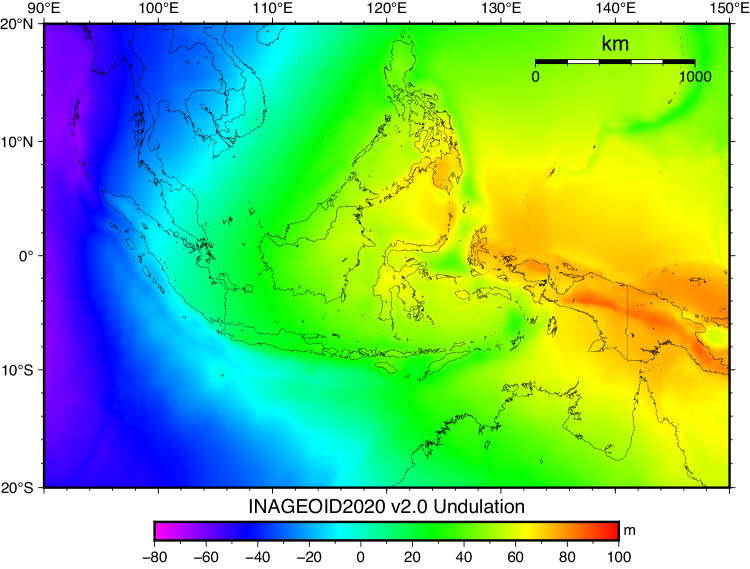
The final INAGEOID2020.

Finally, the final INAGEOID2020 was generated by subtracting the computed geoid with the fitting surface. This geoid model was computed using the Remove-Compute-Restore method, with the EGM2008 geopotential model as the global reference. As the EGM2008 model is defined in the zero tide system, our modeled geoid is consequently also in the zero tide system. Figure 3 depicts Indonesia’s final geoid model, which ranges from −80 to 100 m. For the validation process, this work used geometric geoid data at JKG pillars as the reference to compare its value with the final INAGEOID2020 model. The validation analysis is the subject of the following section.

## Data Records

The processing resulted in a gridded geoid model on the national scale. Based on the results, the geoid in Indonesia ranges from −80 to 100 m, where the east parts generally have higher values than the western. The INAGEOID2020 model developed in this paper is available at 10.5281/zenodo.8404628^49^.

## Technical Validation

Geometric geoid values at JKG pillars were used as reference data. They were calculated based on the difference between geodetic heights from GNSS observations and orthometric heights from levelling measurements. The accuracy of INAGEOID2020 was then computed based on the deviation (∆*N*) between the reference (*N*_*ref*_) and the model (*N*_*INAGEOID*2020_) as in Eq. 4. Based on this metric, the standard deviation (∆*N*_*stdev*_) were calculated to determine the accuracy of INAGEOID2020 since this metric explains the error distribution.

4 $$\triangle N={N}_{{ref}}-{N}_{{INAGEOID}2020}$$

**Fig. 4:**
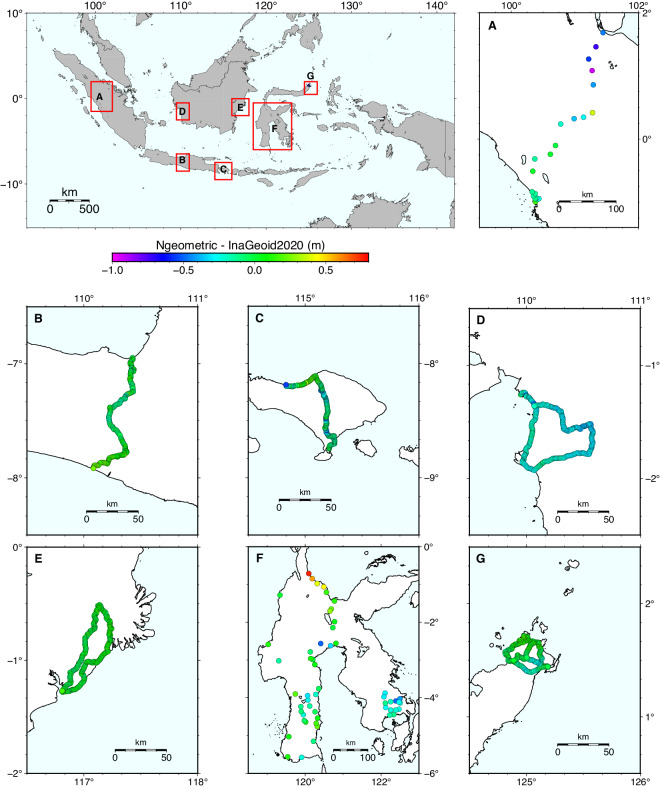
Overview of seven validation areas: Sumatra (**a**) Java (**b**) Bali (**c**) West Kalimantan (**d**) East Kalimantan (**e**) southern Sulawesi (**f**) and northern Sulawesi (**g**). The colored circles represent the difference between geometric and gravimetric height.

Due to the unavailability of reference data in all regions, this work validated seven areas in Sumatra, Java, Bali, Kalimantan, and Sulawesi (Fig. 4). Table 1 depicts the standard deviation of each area. In general, the accuracy of the geoid model varied between approximately 6 cm to 29 cm.

**Table 1:** The validation metrics of INAGEOID2020.

Areas	Number of data	Before Fitting (m)				After Fitting (m)			
		∆*N*_*min*_	∆*N*_*max*_	∆*N*_*mean*_	∆*N*_*stdev*_	∆*N*_*min*_	∆*N*_*max*_	∆*N*_*mean*_	∆*N*_*stdev*_
Java	186	1.769	2.321	2.053	0.122	−0.243	0.301	0.008	0.118
Bali	178	1.768	2.798	2.316	0.200	−0.560	0.367	−0.106	0.167
West Kalimantan	284	1.489	1.958	1.766	0.070	−0.538	−0.001	−0.269	0.064
East Kalimantan	264	1.640	2.160	1.944	0.073	−0.196	0.206	0.020	0.059
South part of Sulawesi	53	1.380	2.655	2.015	0.272	−0.005	0.753	−0.040	0.251
North part of Sulawesi	220	1.673	2.281	1.994	0.116	−0.355	0.270	−0.034	0.121
Sumatra	21	0.881	2.222	1.610	0.293	−0.934	0.372	−0.219	0.286

Model accuracy in Java was computed based on 186 validation points across several regions and cities, including Semarang, Ambarawa, Magelang, Sleman, Yogyakarta, and Kulon Progo, respectively. They started from Semarang tide station and ended at Glagah tide station. Meanwhile, validation points in Bali spread from northern to southern Bali island, starting from Benoa tide station in Badung Regency, Denpasar City, Tabanan Regency, to Celukan Bawang tide station in Buleleng Regency. There were 178 points in total.

On the other hand, the number of validation points in Kalimantan Island was almost twice that of Java and Bali, which were 284 and 264 in West Kalimantan and East Kalimantan, respectively. They were distributed in a loop form. The points in West Kalimantan started from Kayong tide station in Kayong Regency, continued to Ketapang Regency, and returned to Kayong station. In East Kalimantan, they began from Balikpapan tide station in Balikpapan City, continued to Kutai Kartanegara Regency and Samarinda City, and then returned to Balikpapan station.

In contrast, only a few points were available for validation in Sumatra island and the south part of Sulawesi compared to other islands due to a considerable gap between levelling and GNSS measurements, which caused many JKG pillars to become unfeasible. Levelling data was measured around the 1990s, while GNSS data was in the 2010s. The number of points was 53 and 21 in the southern part of Sulawesi and Sumatra, respectively. In Sulawesi, the benchmarks were distributed across the island, from southern to northern South Sulawesi and Southeast Sulawesi regions, with the Ujung Pandang tide station in Makassar City as the reference point. Meanwhile, validation in Sumatra was carried out in the West Sumatra and Riau regions, starting from Padang tide station to Dumai station. Consequently, the model accuracies in Sumatra and south part of Sulawesi are unreliable as in other islands.

New validation points are available within the north part of this island in 2023. A total of 220 points were measured using levelling and GNSS. The resulting standard deviation in this area is more reliable than that of the southern part of Sulawesi. Note that both the geoid undulation model and validation will be updated when new data becomes available.

## Usage Notes

The geoid undulation model developed in this work can be accessed through the SRGI website: https://srgi.big.go.id/map/geoid-active.

### Supplementary information

Geoid Undulation Model as Vertical Reference in Indonesia

## Data Availability

The computations and visualisations presented in this work were done with licensed software Gravsoft software package developed by the DTU Space at the Technical University of Denmark (DTU), Geosoft by Geosoft Inc; AGR software developed by NYCU (National Yang Ming Chiao Tung University); and freely available software QGIS^43^ and GMT^50^. No specific code was used for the creation of this dataset for this research.

## References

[1] Petr V. Why Do We Need a Proper Geoid? *FIG Working Week 2009 - TS3C- GEOID- Modelling* (2009).

[2] Heiskanen, W. A. & Moritz, H. Physical geodesy. *Bulletin Géodésique* **41** (1967).

[3] Hotine, M. *Mathematical Geodesy*. (ESSA Monographs, 1969).

[4] Hofmann-Wellenhof, B. & Moritz, H. *Physical Geodesy*. *Physical Geodesy*10.1007/b139113 (2005).

[5] Vaníček, P., Kingdon, R. & Santos, M. Geoid versus quasigeoid: A case of physics versus geometry. *Contributions to Geophysics and Geodesy* **42** (2012).

[6] Sjöberg, L. E. The geoid or quasigeoid - Which reference surface should be preferred for a national height system? *Journal of Geodetic Science* **3** (2013).

[7] Sideris, M. G. *Geoid Determination - Theory and Principles*. *Encyclopedia of Solid Earth Geophysics*, 10.1007/978-3-540-74700-0 (2011).

[8] Sjöberg, L. E. & Bagherbandi, M. *Gravity Inversion and Integration: Theory and Applications in Geodesy and Geophysics*. *Gravity Inversion and Integration: Theory and Applications in Geodesy and Geophysics*, 10.1007/978-3-319-50298-4 (2017).

[9] Garland, G. D. & Uotila, U. A. Geoid. *Encyclopedia Britannica* https://www.britannica.com/science/geoid (2021).

[10] Hessler, J. Geodesy. in *International Encyclopedia of Human Geography* (eds. Kitchin, R. & Thrift, N.) 390–393 (Elsevier, Oxford, 2009). 10.1016/B978-008044910-4.00028-6.

[11] Osserman, R. Ellipsoid. *Encyclopedia Britannica* https://www.britannica.com/science/ellipsoid (2006).

[12] Prijatna, K. Development of Combination of Gravity and Global Geopotential Model to Determine Regional Geoid in Indonesia Region (in Bahasa Indonesia). (Bandung Institute of Technology, 2010).

[13] Gatchalian, R. C., Forsberg, R. & Olesen, A. V. A new Philippine geoid model from airborne and terrestrial gravity data. *Terrestrial, Atmospheric and Oceanic Sciences* **32** (2021).

[14] Kuczynska-Siehien, J., Lyszkowicz, A. & Birylo, M. Geoid determination for the area of poland by the least squares modification of stokes’ formula. *Acta Geodynamica et Geomaterialia* **13** (2016).

[15] Farahani, H. H., Klees, R. & Slobbe, C. Data requirements for a 5-mm quasi-geoid in the Netherlands. *Studia Geophysica et Geodaetica* **61** (2017).

[16] Hwang, C., Shih, H. C., Hsiao, Y. S. & Huang, C. H. Airborne Gravity Surveys Over Taiwan Island and Strait, Kuroshio Current and South China Sea: Comparison of GPS and Gravity Accuracies at Different Flight Altitudes. *Marine Geodesy* **35** (2012).

[17] Hwang, C. *et al*. Geodetic and geophysical results from a Taiwan airborne gravity survey: Data reduction and accuracy assessment. *J Geophys Res Solid Earth* **112** (2007).

[18] Hwang, C., Hsiao, Y. S. & Shih, H. C. Data reduction in scalar airborne gravimetry: Theory, software and case study in Taiwan. *Comput Geosci* **32** (2006).

[19] Hwang, C. & Hsiao, Y. S. Orthometric corrections from leveling, gravity, density and elevation data: A case study in Taiwan. *J Geod* **77** (2003).

[20] Claessens, S. J., Hirt, C., Amos, M. J., Featherstone, W. E. & Kirby, J. F. The NZGeoid09 model of New Zealand. *Survey Review* **43** (2011).

[21] Amos, M. J. & Featherstone, W. E. Unification of New Zealand’s local vertical datums: Iterative gravimetric quasigeoid computations. *J Geod* **83** (2009).

[22] Dumrongchai, P., Srimanee, C., Duangdee, N. & Bairaksa, J. The determination of Thailand Geoid Model 2017 (TGM2017) from airborne and terrestrial gravimetry. *Terrestrial, Atmospheric and Oceanic Sciences* **32** (2021).

[23] Bramanto, B. *et al*. Determination of gravity anomalies in Java, Indonesia, from airborne gravity survey. *Terrestrial, Atmospheric and Oceanic Sciences* **32** (2021).

[24] Odera, P. A., Fukuda, Y. & Kuroishi, Y. A high-resolution gravimetric geoid model for Japan from EGM2008 and local gravity data. *Earth, Planets and Space* **64** (2012).

[25] Miyahara, B., Kodama, T. & Kuroishi, Y. Development of new hybrid geoid model for Japan, ‘GSIGEO2011’. *Bulletin of the Geospatial Information Authority of Japan* **62** (2014).

[26] Matsuo, K. & Kuroishi, Y. Refinement of a gravimetric geoid model for Japan using GOCE and an updated regional gravity field model. *Earth, Planets and Space* **72** (2020).

[27] Kasenda, A. High Precision Geoid for Modernization of Height Systems in Indonesia. (The Univeristy of New South Wales, 2009).

[28] Khafid, -. On the Unification of Indonesian Local Height Systems. (Technische Universität München, 1997).

[29] Kahar, J., Kasenda, A. & Prijatna, K. The Indonesian Geoid Model 1996. in *Gravity, Geoid and Marine Geodesy* (eds. Segawa, J., Fujimoto, H. & Okubo, S.) 613–620 (Springer Berlin Heidelberg, Berlin, Heidelberg, 1997).

[30] Kahar, J. Geoid Determination in Archipelagic Area. in *Proc. of the General Meeting of IAG Special Issue of J. Geod. Soc, of Japan* (1982).

[31] Prijatna, K. A Strategy for geoid determination in the Indonesian Region. in *DEOS Progress Letters* (ed. Klees, R.) vol. 98, 101–122 (Delft University Press, Delft, 1998).

[32] Pahlevi, A. & Pangastuti, D. Indonesian Geospatial Reference System 2013 and Its Implementation On Positioning. *FIG Congress 2014* **1**, 12 (2014).

[33] Morelli, C. *et al*. *The International Gravity Standardization Net 1971 (I.G.S.N. 71)* (1972).

[34] Wziontek, H. *et al*. Status of the International Gravity Reference System and Frame. *J Geod* **95**, 1–9 (2021). 10.1007/s00190-020-01438-9

[35] Forsberg, R. & Tscherning, C. C. *An Overview Manual for the GRAVSOFT Geodetic Gravity Field Modelling Programs*. (2008).

[36] Sjöberg, L. E. A discussion on the approximations made in the practical implementation of the remove-compute-restore technique in regional geoid modelling. *J Geod* **78** (2005).

[37] Yildiz, H., Forsberg, R., Ågren, J., Tscherning, C. & Sjöberg, L. Comparison of remove-compute-restore and least squares modification of Stokes’ formula techniques to quasi-geoid determination over the Auvergne test area. *Journal of Geodetic Science* **2** (2012).

[38] Abbak, R. A., Erol, B. & Ustun, A. Comparison of the KTH and remove-compute-restore techniques to geoid modelling in a mountainous area. *Comput Geosci* **48** (2012).

[39] Sideris, M. G. Geoid determination by FFT techniques. in *Lecture Notes in Earth System Sciences* vol. 110 (2013).

[40] Pavlis, N. K., Holmes, S. A., Kenyon, S. C. & Factor, J. K. The development and evaluation of the Earth Gravitational Model 2008 (EGM2008). *J Geophys Res Solid Earth* **117** (2012).

[41] Andersen, O. B. & Knudsen, P. The DTU17 global marine gravity field: First validation results. in *International Association of Geodesy Symposia* vol. 150 (2020).

[42] Lestari, R. *et al*. Local geoid modeling in the central part of Java, Indonesia, using terrestrial-based gravity observations. *Geod Geodyn* **14** (2023).

[43] QGIS Development Team. QGIS Geographic Information System. https://qgis.org/en/site/.

[44] Pavlis, N. K., Holmes, S. a., Kenyon, S. C. & Factor, J. K. An earth gravitational model to degree 2160: EGM2008. *presented at the 2008 General Assembly of the European Geosciences Union, Vienna, Austria, April 13-18* **84** (2008).

[45] Lemoine, F. G. *et al*. The Development of the Joint NASA GSFC and the National Imagery and Mapping Agency (NIMA) Geopotential Model EGM96. *NASA Goddard Space Flight Center; Greenbelt, MD United States*. (1998).

[46] Stokes, G. G. On the Effect of the Internal Friction of Fluids on the Motion of Pendulums. in *Mathematical and Physical Papers*10.1017/cbo9780511702266.002 (2010).

[47] Udama, Z. A., Claessens, S., Anjasmara, I. M. & Syafarianty, A. N. Analysis of different combinations of gravity data types in gravimetric geoid determination over Bali. *Journal of Applied Geodesy* **0** (2023).

[48] Forsberg, R. & Tscherning, C. C. *An Overview Manual for the GRAVSOFT Geodetic Gravity Field Modelling Programs*. *Contract Report for JUPEM* (2008).

[49] Pahlevi, A. *et al*. INAGEOID2020: A Vertical Datum in Indonesia, *Zenodo*, 10.5281/zenodo.8404628 (2023). 10.5281/zenodo.8404628

[50] Wessel, P. *et al*. The Generic Mapping Tools Version 6. *Geochemistry, Geophysics, Geosystems* **20** (2019).

