# Supplementary material for: Geoid Undulation Model as Vertical Reference in Indonesia
Source: Sci Data. 2024 Jul 26;11:822. doi: 10.1038/s41597-024-03646-w (PMC11282308; doi:10.1038/s41597-024-03646-w)
Supplement: Supplementary file 1 — Geoid Undulation Model as Vertical Reference in Indonesia [file 41597_2024_3646_MOESM1_ESM.pdf]

Supplementary information to

Geoid Undulation Model as Vertical Reference in Indonesia

Arisauna Pahlevi[1], Agustina Syafarianty[1], Susilo Susilo[2], Yustisi Lumban-Gaol[2], Widy Putra[1], Bagas Triarahmadhana[1], Brian Bramanto[3], Raa Muntaha[1], King El Fadhila[1], Febriananda Ladivanov[1], Harka Amrossalma[1], Lukman Islam[1], Dwi Novianto[1], Safirotul Huda[1], Tunjung Wismadi[1], Joni Efendi[1], Alkindi Ramadhan[1], Dudy Wijaya[3], Kosasih Prijatna[3], Gatot Pramono[1]

[1] Directorate for Geospatial Reference System, Geospatial Information Agency, Indonesia
[2] National Research and Innovation Agency, Indonesia
[3] Geodetic Science, Engineering, and Innovation Research Group, Faculty of Earth Sciences and Technology, Institut Teknologi Bandung, Indonesia

Corresponding author: Susilo Susilo (susilo.2@brin.go.id)

Table of Contents

Figure S1. Terrestrial gravity measurement points in Banda Aceh, Sumatra Island. 2

Figure S2. Terrestrial gravity measurement points in Nias and Medan, Sumatra Island. 2

Figure S3. Terrestrial gravity measurement points in Padang, Riau, and Batam, Sumatra Island. 3

Figure S4. Terrestrial gravity measurement points distribution in Bengkulu, Jambi, Bangka, and Lampung, Sumatra Island. 3

Figure S5. Terrestrial gravity measurement points distribution in Java, Bali, and Lombok islands. 4

Figure S6. Terrestrial gravity measurement points in Kalimantan Island: Pontianak (a), Balikpapan (b), and Tarakan (c). 4

Figure S7. Terrestrial gravity measurement points in Sulawesi Island: Makassar (a), Palu (b), and Manado (c). 4

Figure S8. Terrestrial gravity measurement points in Ambon. 5

Figure S9. Airborne gravity data coverage from the flight line. 5

Figure S10. The area coverage of EGM2008 degree 2190. 6

Figure S11. The area coverage of DTU17 to fill water areas. 6

Figure S12. The distribution of 94 tide gauge benchmarks used for geoid fitting. 7

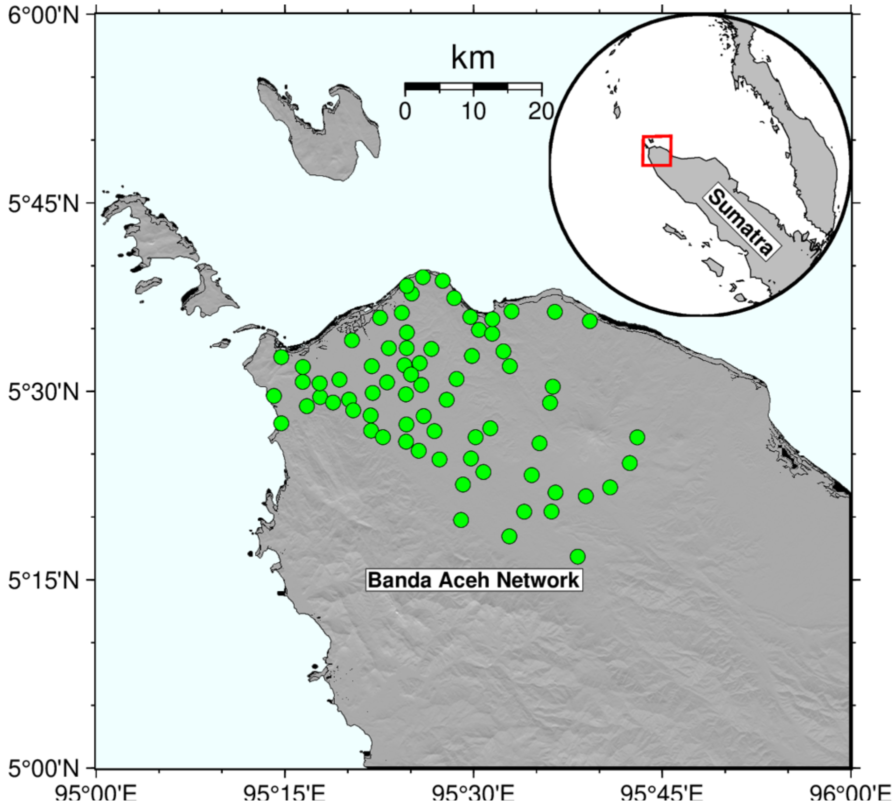

Figure S1. Terrestrial gravity measurement points in Banda Aceh, Sumatra Island.

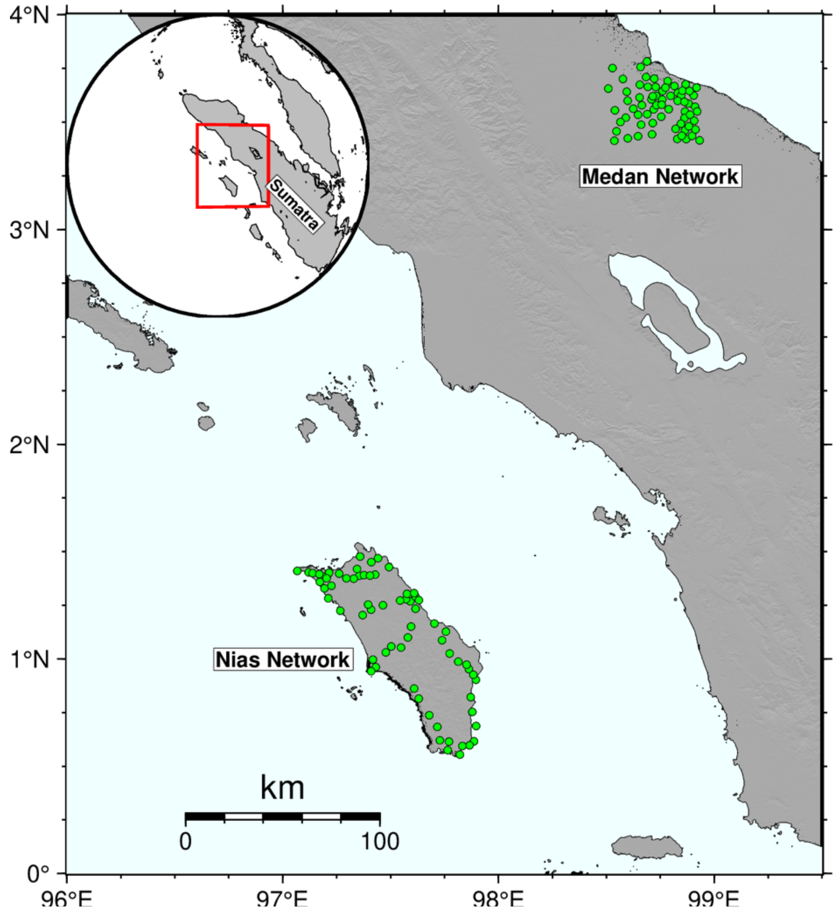

Figure S2. Terrestrial gravity measurement points in Nias and Medan, Sumatra Island.

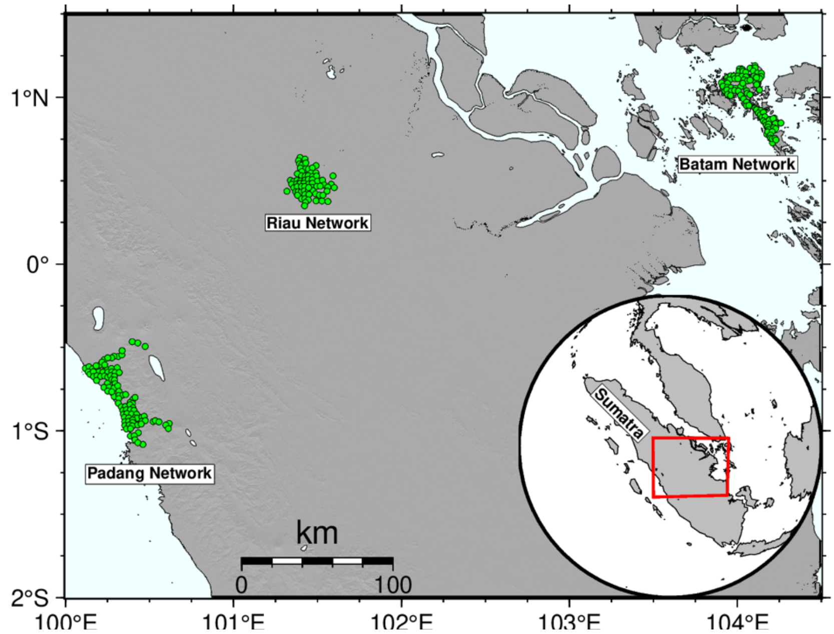

Figure S3. Terrestrial gravity measurement points in Padang, Riau, and Batam, Sumatra Island.

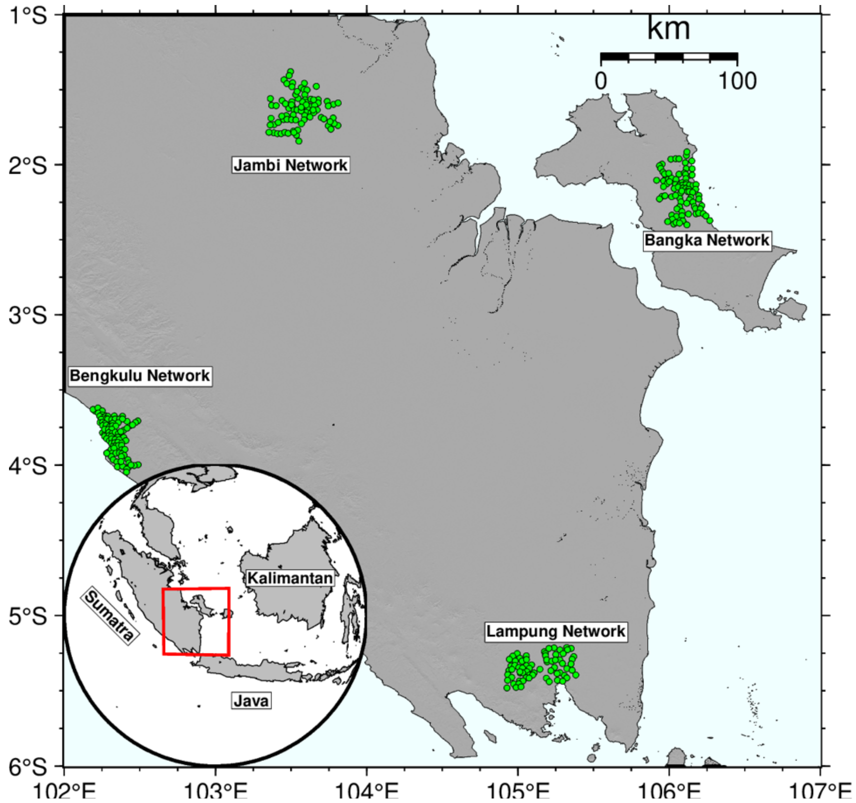

Figure S4. Terrestrial gravity measurement points distribution in Bengkulu, Jambi, Bangka, and Lampung, Sumatra Island.

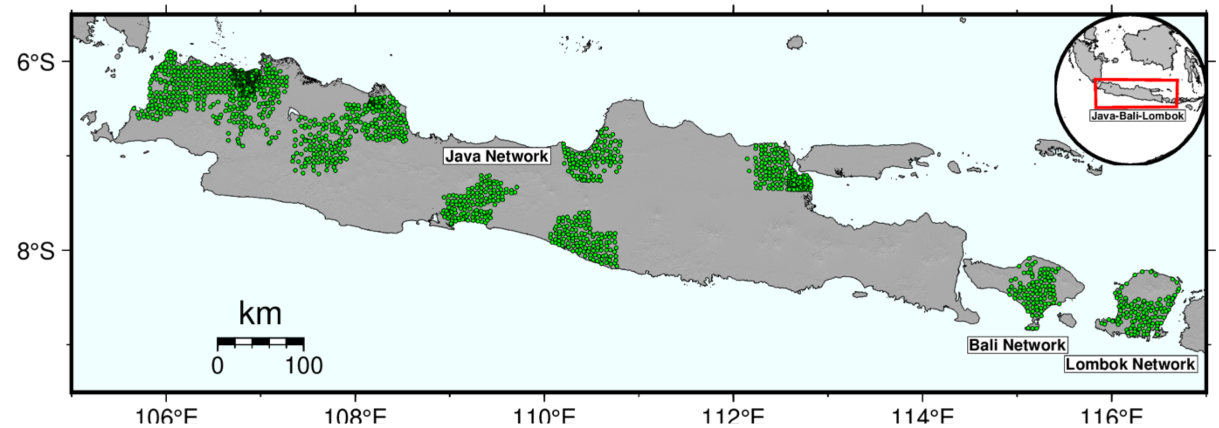

Figure S5. Terrestrial gravity measurement points distribution in Java, Bali, and Lombok islands.

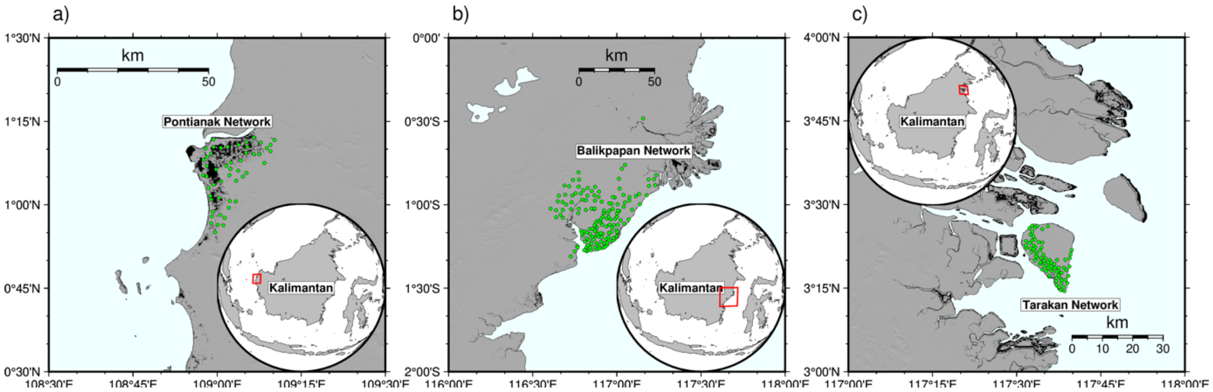

Figure S6. Terrestrial gravity measurement points in Kalimantan Island: Pontianak (a), Balikpapan (b), and Tarakan (c).

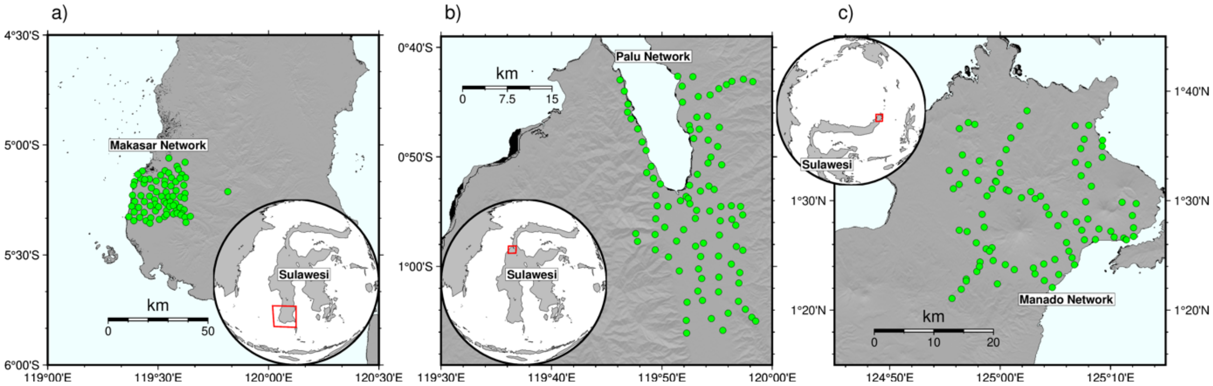

Figure S7. Terrestrial gravity measurement points in Sulawesi Island: Makassar (a), Palu (b), and Manado (c).

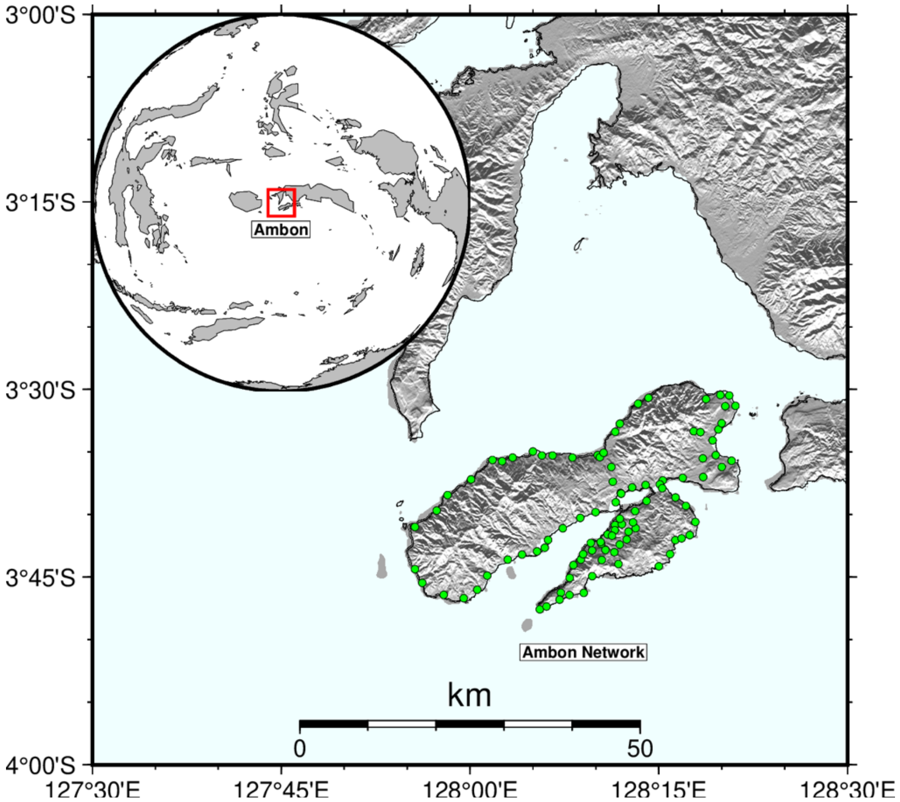

Figure S8. Terrestrial gravity measurement points in Ambon.

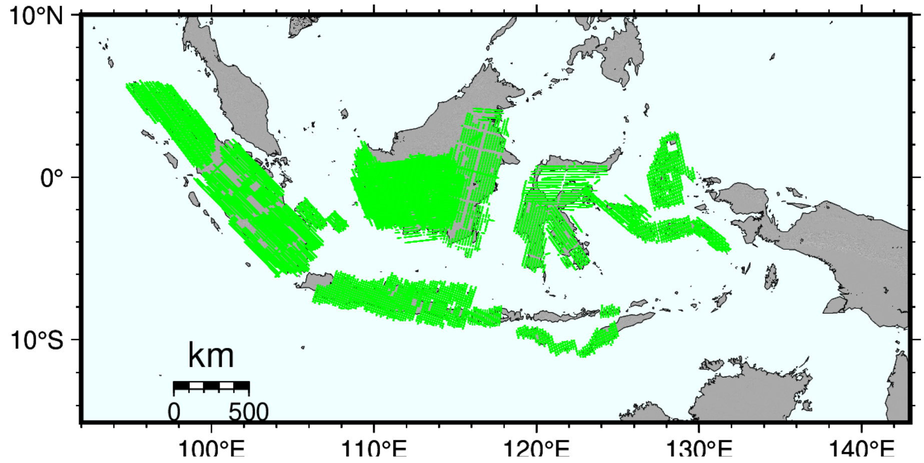

Figure S9. Airborne gravity data coverage from the flight line.

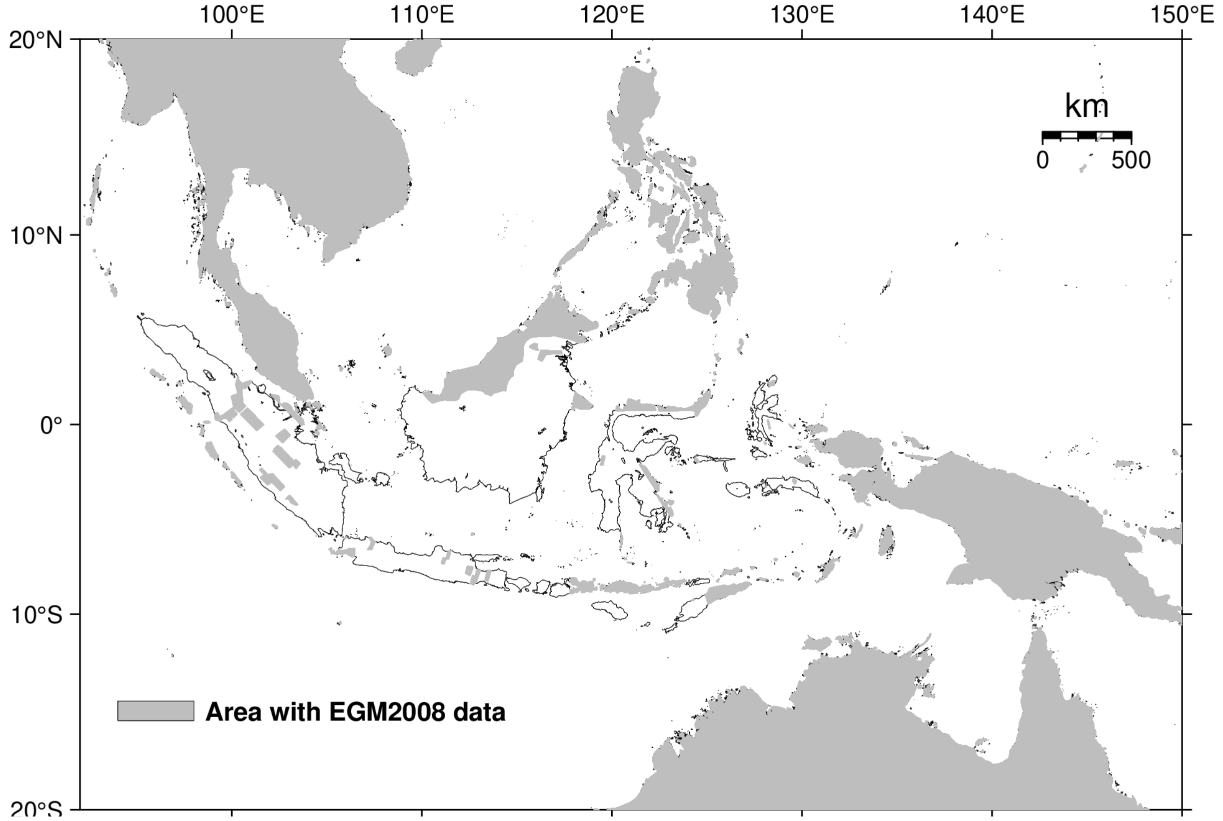

Figure S10. The area coverage of EGM2008 degree 2190.

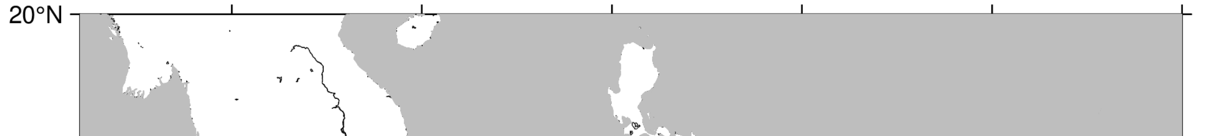

Figure S11. The area coverage of DTU17 to fill water areas.

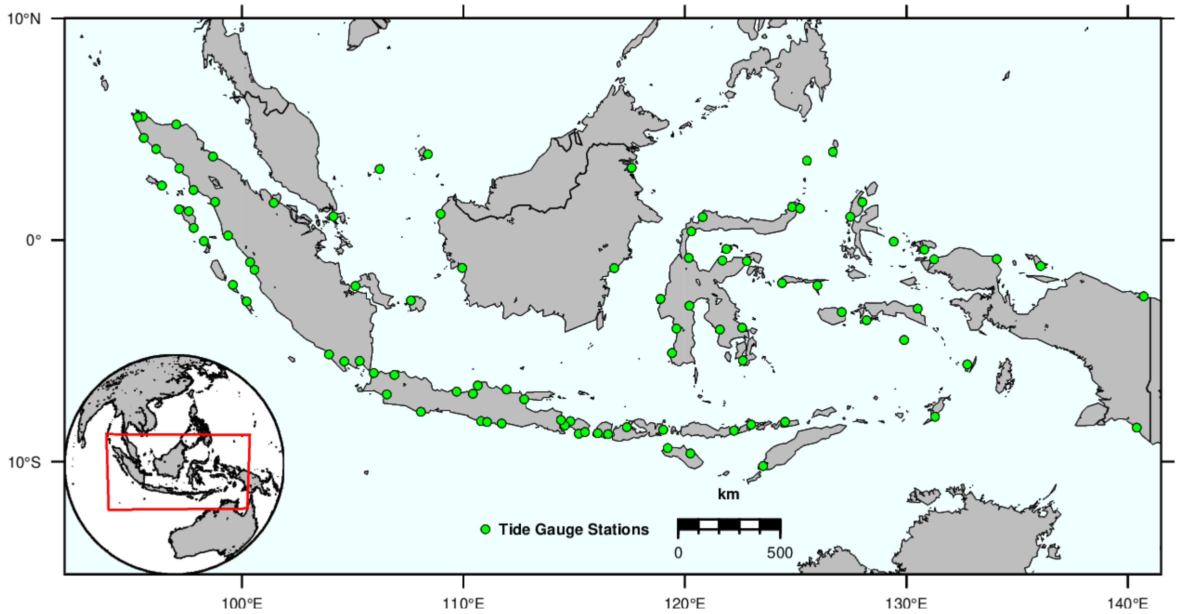

Figure S12. The distribution of 94 tide gauge benchmarks used for geoid fitting.
